# Correlation of a new index reflecting the fluctuation of parasympathetic tone and fetal acidosis in an experimental study in a sheep model

**DOI:** 10.1371/journal.pone.0190463

**Published:** 2018-01-10

**Authors:** C. Garabedian, Y. Clermont-Hama, D. Sharma, E. Aubry, L. Butruille, P. Deruelle, L. Storme, J. De Jonckheere, V. Houfflin-Debarge

**Affiliations:** 1 Univ. Lille, EA 4489 –Perinatal Environment and Health, Lille, France; 2 CHU Lille, Department of Obstetrics, Lille, France; 3 CHU Lille, Department of Pediatric Surgery, Lille, France; 4 CHU Lille, Department of Neonatology, Lille, France; 5 CHU Lille, CIC-IT 1403, Lille, France; University of Minnesota, UNITED STATES

## Abstract

The autonomic nervous system plays a leading role in the control of fetal homeostasis. Fetal heart rate variability (HRV) analysis is a reflection of its activity. We developed a new index (the Fetal Stress Index, FSI) reflecting parasympathetic tone. The objective of this study was to evaluate this index as a predictor of fetal acid-base status. This was an experimental study on chronically instrumented fetal lambs (n = 11, surgery at 128 +/- 2 days gestational age, term = 145 days). The model was based on 75% occlusion of the umbilical cord for a maximum of 120 minutes or until an arterial pH ≤ 7.20 was reached. Hemodynamic, gasometric and FSI parameters were recorded throughout the experimentation. We studied the FSI during the 10 minutes prior to pH samplings and compared values for pH>7.20 and pH≤ 7.20. In order to analyze the FSI evolution during the 10 minutes periods, we analyzed the minimum, maximum and mean values of the FSI (respectively FSI_min_, FSI_max_ and FSI_mean_) over the periods. 11 experimentations were performed. During occlusion, the heart rate dropped with an increase in blood pressure (respectively 160(155–182) vs 106(101–120) bpm and 42(41–45) vs 58(55–62) mmHg after occlusion). The FSI_min_ was 38.6 (35.2–43.3) in the group pH>7.20 and was higher in the group pH less than 7.20 (46.5 (43.3–52.0), p = 0.012). The correlation of FSI_min_ was significant for arterial pH (coefficient of -0.671; p = 0.004) and for base excess (coefficient of -0.632; p = 0.009). The correlations were not significant for the other parameters. In conclusion, our new index seems well correlated with the fetal acid-base status. Other studies must be carried out in a situation close to the physiology of labor by sequential occlusion of the cord.

## Introduction

The monitoring of fetal well being during labor is essentially based on fetal heart rate (FHR) analysis [1]. The recording of FHR, even continuously during labor, does not fully assess fetal oxygenation or neonatal risk [2]. Indeed, this tool is imperfect and subjective with an important inter and intra-operatorvariability [3], despite the existence of classifications [4]. Second-line examinations to characterize the fetal state can be used, i.e. scalp fetal blood sampling to study the fetal acid-base balance (pH or lactates) or study the fetal ECG (ST segment analysis) [5]. These techniques are nevertheless invasive, subject to technical constraints and also STAN is not widespread in North America. There is therefore an important interest in developing both objective and non-invasive means of evaluating fetal well-being.

One of the possibilities studied to better identify fetuses at risk for acidosis is the analysis of changes in the autonomic nervous system (ANS) in response to hypoxia [6,7]. Indeed, the ANS plays a prominent role in the control of fetal homeostasis [8]. Regulation of heart rate is dependent on the ANS and thus, its variability is a reflection of the sympathetic / parasympathetic balance [9]. Spectral analysis is a well-known method of evaluating HRV and high frequency (HF) range (above 0.15 Hz) are centered on the respiratory frequency and related to parasympathetic modulation only [9].

Several publications have studied variations of spectral analysis in fetal acidosis, with conflicting results and a significant individual inter and intra-variability [7,10,11]. In their paper, Casati and Frash pointed out the limitations of the standard spectral approach and concluded that time domain analysis should be preferred [12]. In another study, Frash et al. demonstrated the time domain measure RMSSD (root mean square of successive differences) ability to detect fetal acidemia [13]. We proposed a new continuous tool for the analysis of HRV, the Fetal Stress Index (FSI), taking both advantages of spectral and time domain analysis. Indeed, the FSI uses a spectral analysis (wavelet transform) to filter the signal in order to keep only high frequency oscillations and then computes this oscillations magnitude in the time domain. Our hypothesis is that, because of its reflecting parasympathetic activity, it makes it possible to detect fetuses at risk of acidosis. In a previous study, we shown that this index had a lower inter-subject variability, and a higher effect size compared to other HRV analysis methods [14].

Thus, the aim of this study was to evaluate this new index as a predictive factor of the fetal acid-base state in an experimental model of fetal hypoxia by partial acute occlusion of the cord in pregnant ewes.

## Material and methods

### Ethics

The anesthesia, surgery and experimentation protocols were in line the recommendations of the Ministry of Higher Education and Research and the study was approved by the Animal Experimentation Ethics Committee (CEEA # 2016121312148878).

### Surgical preparation

Eleven near-term pregnant sheep (128 +/- 2 days gestational age, term = 145 days) underwent surgical procedure. Anesthetics and surgical techniques protocols followed those previously described by our team or in the literature [14–16]. Pregnant Charmoise sheep (INRA, Leudeville, France) were fasted 24 h before surgery. Sheep were placed in supine position, intubated, anesthetized with intravenous injection of xylazine (Sedaxylan®, CEVA Santé Animale, France), and maintained with isoflurane 2% (Aerrane®, Baxter, France). The uterus was exteriorized through a maternal midline laparotomy. After hysterotomy, a 4Fr diameter catheter (Arrow®, USA) was inserted into the fetal femoral artery and vein until the abdominal aorta and the inferior vena cava respectively through femoral approach to record gazometric parameters. Electrocardiogram (ECG) electrodes were placed subcutaneously over the chest to record the fetal ECG. An inflatable silicone occluder was placed around the umbilical cord of all fetuses as also a Doppler probe to record cardiac flow. This probe was placed at the same distance as possible for each surgery and helped to adapt occlusion through the inflate occluder to obtain in each case a 75% reduction of the umbilical flow. A 5F5-diameter catheter (Arrow®, USA) was placed into the amniotic cavity for measuring baseline pressure (intra-amniotic pressure, IAP). The fetal arterial catheter and intra-amniotic catheter were connected to pressure sensors (Pressure Monitoring Kit®, Baxter) that were connected to a hemodynamic monitor (monitor Merlin, Helwett Packard, Palo Alto, CA, USA). The mean arterial pressure (MAP) was measured from the blood pressure phasic signal and referenced to the intra-amniotic pressure (IAP). All hemodynamic data and ECG were recorded using the Physiotrace™ data acquisition board with a 1000 Hz sampling rate (Physiotrace™, Estaris Monitoring, Lille, France) [17].

### Calculation of the Fetal Stress Index (FSI)

We previously described the HVR analysis and the algorithm used for the calculation of the FSI [14,18,19]. Briefly, the FSI uses a spectral analysis (wavelet transform) to filter the signal in order to keep only high frequency oscillations and then computes this oscillations magnitude in the time domain. The method consists of detecting each heartbeat from R peak of the ECG signal in order to construct the RR series (time evolution of the time intervals between two heart beats). The RR series is then isolated in a 64 s moving window, normalized and band pass filtered above 0.15 Hz using a wavelet based numerical filter. The remaining oscillations magnitudes are computed by plotting local minima and maxima. The area between the upper and lower envelopes is divided into four subareas A1, A2, A3 and A4. And the AUCmin is defined as the minimum of the 4 subareas. The instantaneous FSI is then computed as:

$$FSI_{inst}=100\times \left(a\times AUC\min+b\right)/12.8$$

where a and b are two constants determined on a 200 patients dataset in order to keep the coherence between the visual aspect and the quantitative measurement of FSI. The FSI is then computed as the average value of FSI_inst_ over the last 3 minutes.

### Experimental procedure

The experiments were starting only after a 48-hour rest of the sheep. During this period, 0,3mL/10kg de Buprénorphine were daily injected to ensure post op analgesia. Before occlusion, a first 30-minute period (stability period) was recorded to ensure that the animals were healthy and representative of the model (normal blood gases and stable hemodynamic prameters). The occlusion was done to obtain a 75% reduction of umbilical flow (controlled with the umbilical probe) and lasted until measurement of a pH ≤ 7.20 or a maximum duration of 120 mn. Hemodynamic parameters (heart rate, mean blood pressure, intra-amniotic pressure, flow of the umbilical artery) and blood gazes were recorded every 15 minutes. Ewes were not restrained or sedated during the experimentation. After the experiment or in case of miscarriage or fetal death, the animals were sacrified by an injection of embutramide (T61®, Intervet, France).

### Statistical analysis

In order to validate the fetal hypoxia model, we first compared hemodynamic and gazometric parameters between periods before occlusion and 15, 30 and 45 minutes after occlusion. Comparisons between time periods were performed using the Friedman non-parametric test. Significance threshold was set at P <0.05.

To homogenize the results, we chose to present the results by pH category. The thresholds of 7,20 was retained in accordance with those already used in the literature and the clinical implication of such a pH when performing a fetal blood sample [20]. The data of the different parameters during occlusion were compared between pH> 7.20 and pH ≤7.20. The importance of the early detection of fetal hypoxia being able to intervene before the occurrence of asphyxia, and thus avoid possible neonatal brain sequelae, we chose to study the FSI evolution during the 10 minutes prior to pH samplings. We therefore analyzed the minimum, maximum and mean values of the FSI (respectively FSI_min_, FSI_max_ and FSI_mean_) over these periods.

Hemodynamic, gazometric parameters and the FSI_min_, FSI_max_ and FSI_mean_ were expressed as median and interquartile range (1^st^ quartile-3rd quartile). Comparisons between pH>7.20 and pH≤7.20 were performed using the Wilcoxon non-parametric test. Significance threshold was set at P <0.05. The relationships between gazometric parameters (pH, pO2, pCO2, lactates and base deficit) and FSI values were evaluated using the Spearman Rho correlation coefficients. The software used was SPSS 20.0 (IBM, Armonk, NY, USA).

## Results

11 experimental procedures were performed and 3 were excluded for unusable datas (rapid fall of pH in 2 cases, no gazometric datas in one case due to a thrombus the arterial catheter). No miscarriage or fetal death occurred. Table 1 shows the gazometric and hemodynamic datas time evolution. Before occlusion, ewes were healthy with a normal (pH = 7.40). At T = 15mn, we observed a bradychardia (106 bpm vs 160 before occlusion) and an hypertension (58 vs. 42 mmHg). Gazometric parameters showed a median pH of 7.23 with an hypoxemia (pO2 = 11.5mmHg) and a rise of lactates, pCO2 and base deficit. All pH were superior to 7.20. At T = 30mn, hemodynamic parameters were stable. In 6 experimentations, pH was less than 7.20. At T = 45mn, median pH was 7.12 and all were inferior to 7.20. We observed metabolic acidosis with lactates at 5.83 mmol/l and base deficit at 11 mmol/l.

**Table 1:** Physiological fetal datas in time series (n = 8).

	Before occlusion	After occlusion			p
		T15mn	T30mn	T45mn	
Heart rate (bpm)	160(155–182)	106(101–120)*	103(94–122)*	99(89–127)*	0.002
Art pressure (mmHg)	42(41–45)	58(55–62)*	57(54–59)*	59(55–65)*	0.002
pH	7.4(7.4–7.4)	7.23(7.22–7.25)*	7.18(7.11–7.20)*	7.12(7.08–7.14)*	<0.0001
pO_2_ (mmHg)	16(15–18.25)	11.5(10.25–12.75)*	13(11.25–13.75)*	13(12–14)*	0.002
pCO_2_ (mmHg)	36.1(29.7–39.7)	48.1(40.2–57.9)*	53.7(41.3–64.5)*	59.4(55.9–64.9)*	0.002
Lactates (mmol/l)	0.89(0.76–1.38)	2.57(1.94–3.62)*	4.47(3.06–5.40)*	5.83(5.04–6.61)*	<0.0001
Base deficit (mmol/l)	3.5(0–6.75)	6.5(2.25–9.75)*	7.5(4–13.75)*	11(9–11)*	0.026

Results presented as median and interquartile. p values represent the comparison beetween the four periods (Friedman test).

* represents the comparison the occlusion period and the baseline (Wilcoxon test).

Table 2 shows hemodynamic, gazometric and FSI variations according to the pH ranges during occlusion. Lactates were significantly higher for pH≤7.20 (p = 0.012) whereas other gazometric parameters didn’t vary significantly. Heart rate and arterial pressure remained the same between pH>7.20 and pH≤7.20.

**Table 2:** FSI variation according to pH ranges during occlusion.

	pH>7.20	pH≤7.20	p
Heart rate (bpm)	106(101–120)	106(97–129)	0.499
Arterial pressure (mmHg)	57.5(55.25–62.25)	57(55.5–64.25)	1.000
pH	7.23(7.22–7.27)	7.17(7.14–7.18)	0.012
pO_2_ (mmHg)	11.5(10.25–12.75)	13(11.25–13.75)	0.356
pCO_2_ (mmHg)	48.1(40.2–57.9)	50.8(38.9–63.6)	1.000
Lactates (mmol/l)	2.57(1.94–3.62)	4.47(2.92–5.57)	0.012
Base deficit (mmol/l)	6.5(2.25–9.75)	9.5(6.25–14.5)	0.058
FSI_min_	38.6(35.2–43.3)	46.5(43.3–52)	0.012
FSI_max_	64.6(61.4–74.9)	67.7(58.7–73.2)	0.779
FSI_mean_	52.1(47.8–59.4)	56.4(51.9–63.2)	0.123

Results presented as median and interquartile. p values represent the comparison beetween the two groups (Wilcoxon test).

**Fig 1:**
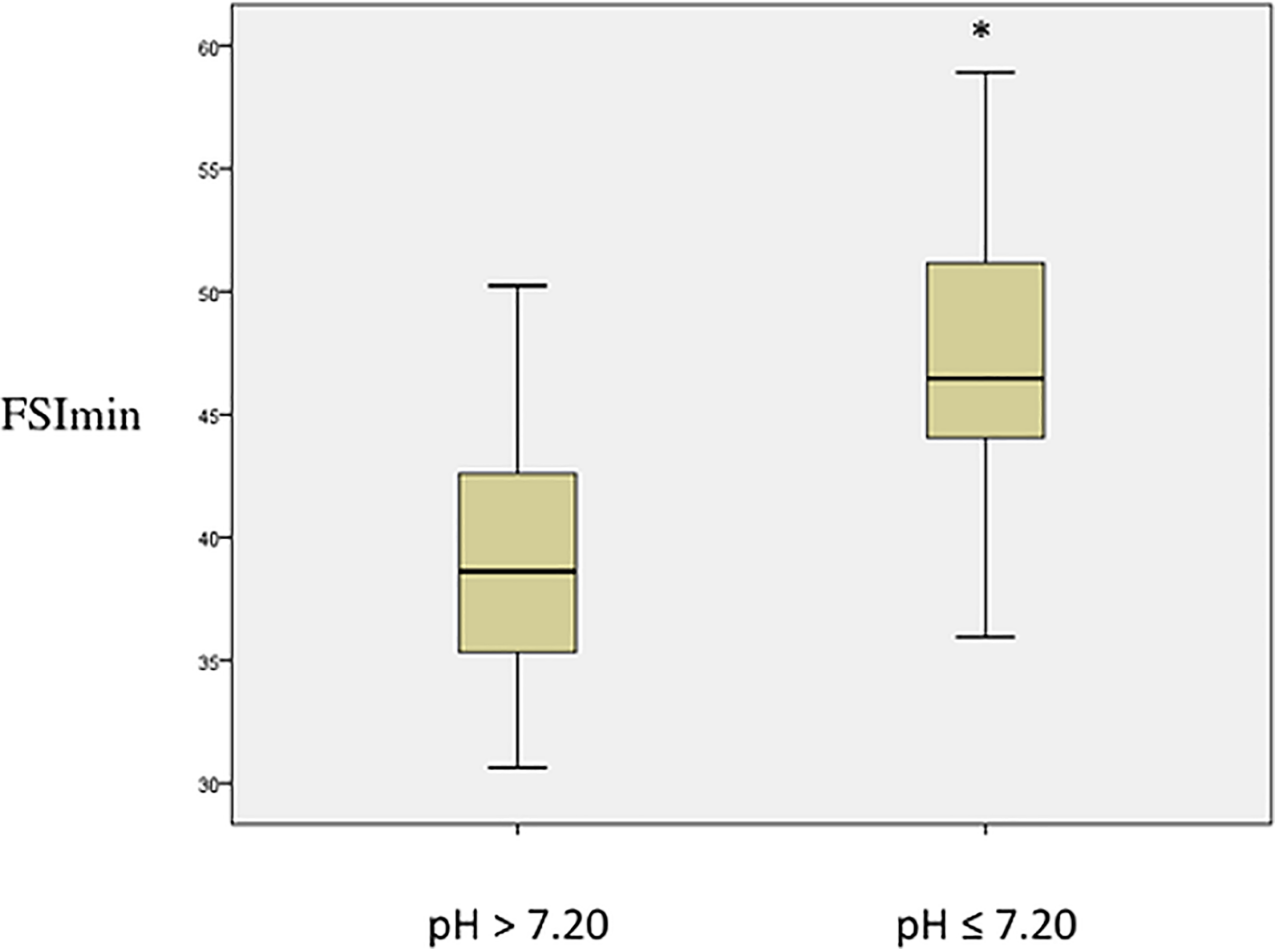
Fetal Stress Index according to pH. FSI_min_ according to pH during occlusion (n = 8). The data are presented as median ± interquartile. *: p<0.05 versus pH> 7.20.

**Fig 2:**
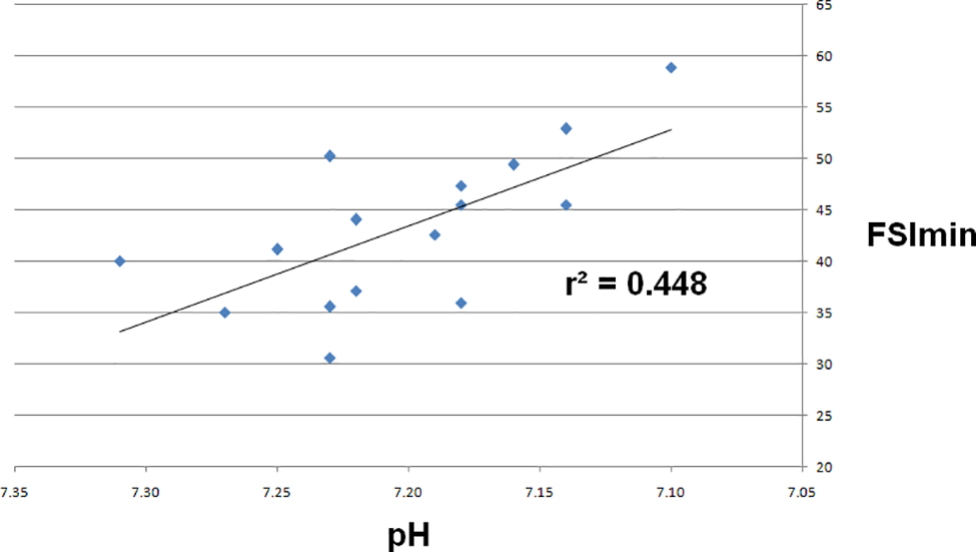
Correlation curve between FSI_min_ and arterial pH.

**Fig 3:**
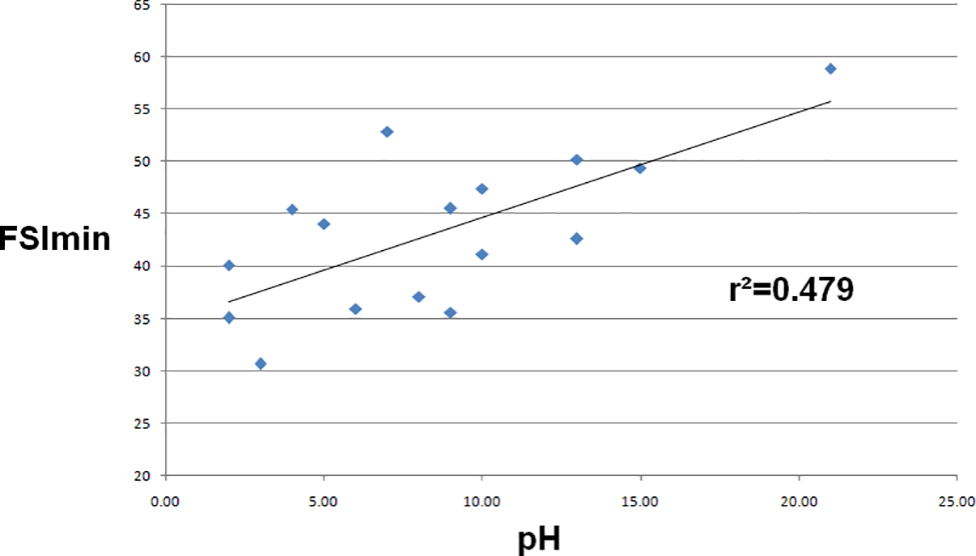
Correlation curve between FSI_min_ and base deficit.

The FSI_min_ was 38.6 (35.2–43.3) for pH>7.20 and was higher for pH≤7.20 (46.5 (43.3–52.0), p = 0.012) (Fig 1). FSI_max_ and FSI_mean_ didn’t show significant variations between pH≤7.20 and pH>7.20 (Table 2). The correlation between FSI_min_ and hemodynamic—gasometric parameters is shown in Table 3. The correlation was significant for arterial pH (coefficient of -0.671; p = 0.004) (Fig 2), and for base deficit (coefficient of -0.632; p = 0.009) (Fig 3). The correlations were not significant for the other parameters.

**Table 3:** Correlation between hemodynamic, gazometric parameters and FSI_min_ during occlusion.

Gazometric parameter	AP	HR	pH	pO_2_	pCO_2_	Lact.	BD
Correlation coefficient (r values)	0.368	0.0049	-0.671	0.142	0.311	0.024	0.63
p	0.161	0.858	0.004	0.60	0.24	0.0931	0.009

HR: Heart rate; AP: Arterial pressure; Lact.: Lactates; BD: Base deficit

For the 2 cases excluded due to rapid fall of pH, fetal sheep were hypoxemic before occlusion with baseline p0_2_ values lowers than 15 mmHg.

## Discussion

The aim of fetal monitoring during labor is to detect fetuses at risk of developing respiratory and metabolic acidosis, which could result in brain lesions or even perpartum death [21,22]. This monitoring is based primarily on the continuous recording of the FHR, which does not, however, allow a perfect evaluation of oxygenation of the fetus and whose limits of interpretation are well known. The current second-line examinations are invasive and are only feasible under certain conditions, with controversial efficacy [23,24]. The objective of this experimental study on fetal lamb was to evaluate a new index based on the analysis of the parasympathetic ANS activity through HRV. Our results showed a good correlation between pH and FSI and that this index made it possible to predict the occurrence of moderate acidosis.

In order to obtain acidosis, we chose a model of chronically instrumented fetal lambs with occlusion of the umbilical cord. Several experimental models of fetal hypoxia are found in the literature, particularly in fetal lamb: partial occlusion of the maternal aorta, occlusion of uterine or hypogastric arteries, repeated umbilical cord total occlusions of varying duration and frequency, or maternal hypoxemia by oxygen depletion of the air inspired by the mother [25,26]. In our study, we chose to achieve partial, acute and prolonged occlusion of the umbilical artery (reduction of 75% of the flow) in order to obtain a gradual decrease of the pH allowing the analysis of the phenomena of fetal adaptation brought into play. Indeed, occlusion was responsible for a fall in heart rate and an increase in mean arterial pressure. Several studies have shown that bradycardia and peripheral vasoconstriction were secondary to activation of the carotid receptor block after sinocarotidial and sinoaortic denervation [27,28]. Indeed, the reduction in the size of the common umbilical artery during occlusion is mechanically responsible for an increase in peripheral vascular resistance and in mean arterial pressure [29]. This increase stimulates baroreceptors in the carotid sinus and aorta, resulting in a vagus-mediated parasympathetic response that decreases heart rate [30]. However, the role of baroreflex is discussed by some teams [8]. Indeed formal studies sheep confirm that the baroreflexes are immature before birth [8,31]. Thus, modern interpretation is that the baroreflex makes only a limited contribution to fetal adaptation to asphyxia and the main actor is the chemoreflex.

We carried out an original analysis of the HRV based both on spectral and time analysis. Indeed, the FSI uses a spectral analysis to filter the signal in order to keep only high frequency oscillations and then computes these oscillations magnitudes in the time domain. At present, there is no standardized method for analyzing fetal HRV and different techniques have been proposed: time analysis with the RR interval study or spectrum analysis of heart rate fluctuation frequencies using the Fourier or Wavelet transform [6,13,32,33]. In the literature, some studies have separated in high from low frequencies spectral content, while others studied the total spectral band [6,7,10]. Thus, there is as yet neither method currently used in routine of HRV analysis nor any clinical reference analysis.

In our experimental study with continuous acute occlusion of the cord and outside the labor period, we observed an increase in our index as soon as moderate acidosis occurred (from 7.20). The study of the correlations showed that our index was correlated to the variation of the pH and with the base deficit. Other studies have investigated changes in HRV by spectral analysis in acidosis situations [6,7,10]. These studies collected the ECG signal from a fetal scalp electrode and proposed an a posteriori comparison of HRV, either with respect to scalp pH or with cord pH at birth. Siira, et al. carried out the analysis of the correlation between HRV and the pH value during labor (scalp pH) on 543 fetuses [6]. They found an increase in total HRV in the fetus group with scalp pH <7.20 (81 fetuses) compared to those with a pH ≥7.20 (462 fetuses). However, in the subgroup of fetuses with a pH <7.10, HRV was similar to that of normoxic fetuses. Furthermore, Van Laar, et al. found no correlation between HRV in high or low frequencies in the human fetus during labor for scalp pHs between 7.20 and 7.25 in case of absolutes HRV parameters (study on 39 pH in utero in 30 patients, signal collected by scalp electrode) [34]. However, after normalizing the values (division of LF and HF power by the total power), they found an increase in the normalized HF and a decrease in normalized LF when the intrauterine pH decreased. In another study analyzing a posteriori RCF recordings 1 h before the birth of neonates with severe acidosis compared to neonates with a pH > 7.20, Van Laar, et al. reported an increase LF and a decrease HF within 30 minutes before birth in the newborn group with a pH <7.05 compared to the others [7]. In a first pilot retrospective study, we also found a decrease of our index in case of pH less than 7.15 during second stage of labor [19].

Thus, divergent results are found on the variations of HRV according to the studies, probably due to techniques (measurement of the total HRV, normalization of the LF and the HF…) but also of different measurement conditions (scalp sample or neonatal pH). Our index appears promising because of its ease of interpretation (numerical data), its continuous character and its ability to detect situations of moderate hypoxia. It could therefore be a warning sign for acidosis.

However, this experimental study has several limitations. The first one is that of being an animal model. Indeed, despite many similarities between sheep and human gestation, the reproducibility of our physiopathological observations and therefore the extrapolation to the human fetus must therefore be considered with caution. Our model can be also discussed. It is not like a labor insult, neither a clinical scenario, but we aim to evaluate the variation in case of acute hypoxia as proposed by many other authors [35–37]. It would be interesting to modify our model to approximate the physiology of labor through sequential occlusions that recall the effect of uterine contractions as described by several authors [22,38,39]. It will be also interesting to evaluate the use of nitrogen to lower maternal and thus fetal pO2 in a controlled manner without effecting fetal perfusion. At last, we observed only 48h of rest after surgery and fetal pO2 at the start of the experiments was low and possibly reflected the effects of the surgical procedure. It could be considered moderately hypoxic and it will be important in future projects to ensure a longer period of recovery.

## Conclusion

In this experimental study, our new index seems well correlated with the fetal acid-base status. Other studies must be carried out in a situation close to the physiology of labor by sequential occlusion of the cord.

## References

[1] Everett TR, Peebles DM. Antenatal tests of fetal wellbeing. Semin Fetal Neonatal Med. 2015 6;20(3):138–43. doi: 10.1016/j.siny.2015.03.011 25936927 10.1016/j.siny.2015.03.011

[2] Clark SL, Hamilton EF, Garite TJ, Timmins A, Warrick PA, Smith S. The limits of electronic fetal heart rate monitoring in the prevention of neonatal metabolic acidemia. Am J Obstet Gynecol. 2016 10 14; 10.1016/j.ajog.2016.10.009 27751795

[3] Ayres-de-Campos D, Spong CY, Chandraharan E. FIGO consensus guidelines on intrapartum fetal monitoring: Cardiotocography. Int J Gynecol Obstet. 2015 10;131(1):13–24. 10.1016/j.ijgo.2015.06.020 26433401

[4] Blackwell SC, Grobman WA, Antoniewicz L, Hutchinson M, Gyamfi Bannerman C. Interobserver and intraobserver reliability of the NICHD 3-Tier Fetal Heart Rate Interpretation System. Am J Obstet Gynecol. 2011 10;205(4):378.e1–378.e5. 21864826 10.1016/j.ajog.2011.06.086

[5] Visser GH, Ayres-de-Campos D. FIGO consensus guidelines on intrapartum fetal monitoring: Adjunctive technologies. Int J Gynecol Obstet. 2015 10;131(1):25–9. 10.1016/j.ijgo.2015.06.021 26433402

[6] Siira SM, Ojala TH, Vahlberg TJ, Rosén KG, Ekholm EM. Do spectral bands of fetal heart rate variability associate with concomitant fetal scalp pH? Early Hum Dev. 2013 9;89(9):739–42. doi: 10.1016/j.earlhumdev.2013.05.007 23809772 10.1016/j.earlhumdev.2013.05.007

[7] Van Laar J, Peters C, Vullings R, Houterman S, Bergmans J, Oei S. Fetal autonomic response to severe acidaemia during labour. BJOG Int J Obstet Gynaecol. 2010 3 1;117(4):429–37. 10.1111/j.1471-0528.2009.02456.x 20025619

[8] Lear CA, Galinsky R, Wassink G, Yamaguchi K, Davidson JO, Westgate JA, Bennet L, Gunn AJ. The myths and physiology surrounding intrapartum decelerations: the critical role of the peripheral chemoreflex. J Physiol. 2016 9 1;594(17):4711–25. doi: 10.1113/JP271205 27328617 10.1113/JP271205 PMC5009777

[9] Akselrod S, Amitayt Y, Lang RM, Mor-Avi V, Keselbrener L. Spectral analysis of left ventricular area variability as a tool to improve the understanding of cardiac autonomic control. Physiol Meas. 2000 5;21(2):319–31. 10847198 10.1088/0967-3334/21/2/311

[10] Siira SM, Ojala TH, Vahlberg TJ, Jalonen JO, Välimäki IA, Rosén KG, Ekholm EM. Marked fetal acidosis and specific changes in power spectrum analysis of fetal heart rate variability recorded during the last hour of labour. BJOG Int J Obstet Gynaecol. 2005 4;112(4):418–23. 10.1111/j.1471-0528.2004.00454.x 15777438

[11] Chung DY, Sim YB, Park KT, Yi SH, Shin JC, Kim SP. Spectral analysis of fetal heart rate variability as a predictor of intrapartum fetal distress. Int J Gynaecol Obstet Off Organ Int Fed Gynaecol Obstet. 2001 5;73(2):109–16. 10.1016/s0020-7292(01)00348-4 11336729

[12] Casati D, Frasch MG. Analysis of fetal heart rate variability in frequency domain: methodical considerations. Exp Physiol. 2014 2;99(2):466–7. doi: 10.1113/expphysiol.2013.076539 24487249 10.1113/expphysiol.2013.076539 PMC5359011

[13] Frasch MG, Müller T, Weiss C, Schwab K, Schubert H, Schwab M. Heart rate variability analysis allows early asphyxia detection in ovine fetus. Reprod Sci Thousand Oaks Calif. 2009 5;16(5):509–17. 10.1177/1933719108327597 19164481

[14] Garabedian C, Champion C, Servan-Schreiber E, Butruille L, Aubry E, Sharma D, Logier R, Deruelle P, Storme L, Houfflin-Debarge V, Jonckheere JD. A new analysis of heart rate variability in the assessment of fetal parasympathetic activity: An experimental study in a fetal sheep model. PLOS ONE. 2017 Juil;12(7):e0180653 doi: 10.1371/journal.pone.0180653 28700617 10.1371/journal.pone.0180653 PMC5503275

[15] Aubry E, Fayoux P, Jani J, Deprest J, Deruelle P, Houfflin-Debarge V, Storme L. Tracheal occlusion alters pulmonary circulation in the fetal lamb with normally developing lungs. J Pediatr Surg. 2013 3;48(3):481–7. doi: 10.1016/j.jpedsurg.2012.08.024 23480900 10.1016/j.jpedsurg.2012.08.024

[16] Houfflin-Debarge V, Sabbah-Briffaut E, Aubry E, Deruelle P, Alexandre C, Storme L. Effects of environmental tobacco smoke on the pulmonary circulation in the ovine fetus. Am J Obstet Gynecol. 2011 5;204(5):450.e8–450.e14. 10.1016/j.ajog.2010.12.049 21333966

[17] De Jonckheere J, Logier R, Dassonneville A, Delmar G, Vasseur C. PhysioTrace: An efficient toolkit for biomedical signal processing. Conf Proc Annu Int Conf IEEE Eng Med Biol Soc IEEE Eng Med Biol Soc Annu Conf. 2005;7:6739–41. 10.1109/IEMBS.2005.1616051 17281820

[18] Garabedian C, Butruille L, Servan-Schreiber E, Ficheur G, Storme L, Deruelle P, De Jonckheere J, Houfflin-Debarge V. Fetal Heart-Rate Variability: Validation of a New Continuous, Noninvasive Computerized Analysis. Gynecol Obstet Invest. 2016 12 14; 10.1159/000452668 27960173

[19] Butruille L, De Jonckheere J, Flocteil M, Garabedian C, Houfflin-Debarge V, Storme L, Deruelle P, Logier R. Parasympathetic tone variations according to umbilical cord pH at birth: a computerized fetal heart rate variability analysis. J Clin Monit Comput. 2016 11 15; 10.1007/s10877-016-9957-y 27848142

[20] Carbonne B, Pons K, Maisonneuve E. Foetal scalp blood sampling during labour for pH and lactate measurements. Best Pract Res Clin Obstet Gynaecol. 2016 1;30:62–7. doi: 10.1016/j.bpobgyn.2015.05.006 26253238 10.1016/j.bpobgyn.2015.05.006

[21] Amaya KE, Matushewski B, Durosier LD, Frasch MG, Richardson BS, Ross MG. Accelerated acidosis in response to variable fetal heart rate decelerations in chronically hypoxic ovine fetuses. Am J Obstet Gynecol. 2016 2;214(2):270.e1–270.e8. 26433172 10.1016/j.ajog.2015.09.084

[22] Frasch MG, Mansano RZ, Gagnon R, Richardson BS, Ross MG. Measures of acidosis with repetitive umbilical cord occlusions leading to fetal asphyxia in the near-term ovine fetus. Am J Obstet Gynecol. 2009 2;200(2):200.e1–200.e7. 19111277 10.1016/j.ajog.2008.10.022

[23] Chandraharan E. Fetal scalp blood sampling should be abandoned: FOR: FBS does not fulfil the principle of first do no harm. BJOG Int J Obstet Gynaecol. 2016 10 1;123(11):1770–1770. 10.1111/1471-0528.13980 27653325

[24] Belfort MA, Saade GR, Thom E, Blackwell SC, Reddy UM, Thorp JM, Tita ATN, Miller RS, Peaceman AM, McKenna DS, Chien EKS, Rouse DJ, Gibbs RS, El-Sayed YY, Sorokin Y, Caritis SN, VanDorsten JP. A Randomized Trial of Intrapartum Fetal ECG ST-Segment Analysis. N Engl J Med. 2015 8 13;373(7):632–41. doi: 10.1056/NEJMoa1500600 26267623 10.1056/NEJMoa1500600 PMC4631435

[25] Bennet L, Peebles DM, Edwards AD, Rios A, Hanson MA. The cerebral hemodynamic response to asphyxia and hypoxia in the near-term fetal sheep as measured by near infrared spectroscopy. Pediatr Res. 1998 12;44(6):951–7. doi: 10.1203/00006450-199812000-00022 9853934 10.1203/00006450-199812000-00022

[26] Castillo-Melendez M, Baburamani AA, Cabalag C, Yawno T, Witjaksono A, Miller SL, Walker DW. Experimental Modelling of the Consequences of Brief Late Gestation Asphyxia on Newborn Lamb Behaviour and Brain Structure. Rogers LK, editor. PLoS ONE. 2013 11 6;8(11):e77377 doi: 10.1371/journal.pone.0077377 24223120 10.1371/journal.pone.0077377 PMC3819360

[27] Jensen A, Garnier Y, Berger R. Dynamics of fetal circulatory responses to hypoxia and asphyxia. Eur J Obstet Gynecol Reprod Biol. 1999 6;84(2):155–72. 10428339 10.1016/s0301-2115(98)00325-x

[28] Jensen A, Hanson MA. Circulatory responses to acute asphyxia in intact and chemodenervated fetal sheep near term. Reprod Fertil Dev. 1995;7(5):1351–9. 8848611 10.1071/rd9951351

[29] Booth LC, Malpas SC, Barrett CJ, Guild S-J, Gunn AJ, Bennet L. Is baroreflex control of sympathetic activity and heart rate active in the preterm fetal sheep? Am J Physiol—Regul Integr Comp Physiol. 2009 3 1;296(3):R603–9. doi: 10.1152/ajpregu.90624.2008 19109368 10.1152/ajpregu.90624.2008

[30] Pinas A, Chandraharan E. Continuous cardiotocography during labour: Analysis, classification and management. Best Pract Res Clin Obstet Gynaecol. 2016 1;30:33–47. doi: 10.1016/j.bpobgyn.2015.03.022 26165747 10.1016/j.bpobgyn.2015.03.022

[31] Galinsky R, Lear CA, Yamaguchi K, Wassink G, Westgate JA, Bennet L, Gunn AJ. Cholinergic and β-adrenergic control of cardiovascular reflex responses to brief repeated asphyxia in term-equivalent fetal sheep. Am J Physiol Regul Integr Comp Physiol. 2016 9 21;ajpregu.00340.2016. 10.1152/ajpregu.00340.2016 27654399

[32] Durosier LD, Green G, Batkin I, Seely AJ, Ross MG, Richardson BS, Frasch MG. Sampling rate of heart rate variability impacts the ability to detect acidemia in ovine fetuses near-term. Front Pediatr. 2014;2:38 doi: 10.3389/fped.2014.00038 24829897 10.3389/fped.2014.00038 PMC4017161

[33] Van Laar J o. e. h., Porath M m., Peters C h. l., Oei S g. Spectral analysis of fetal heart rate variability for fetal surveillance: review of the literature. Acta Obstet Gynecol Scand. 2008 3 1;87(3):300–6. doi: 10.1080/00016340801898950 18307069 10.1080/00016340801898950

[34] Laar JOEH van, Peters CHL, Houterman S, Wijn PFF, Kwee A, Oei SG. Normalized spectral power of fetal heart rate variability is associated with fetal scalp blood pH. Early Hum Dev. 2011 4 1;87(4):259–63. doi: 10.1016/j.earlhumdev.2011.01.028 21316165 10.1016/j.earlhumdev.2011.01.028

[35] Koome ME, Davidson JO, Drury PP, Mathai S, Booth LC, Gunn AJ, Bennet L. Antenatal dexamethasone after asphyxia increases neural injury in preterm fetal sheep. PloS One. 2013;8(10):e77480 doi: 10.1371/journal.pone.0077480 24204840 10.1371/journal.pone.0077480 PMC3799621

[36] Yawno T, Mahen M, Li J, Fahey MC, Jenkin G, Miller SL. The Beneficial Effects of Melatonin Administration Following Hypoxia-Ischemia in Preterm Fetal Sheep. Front Cell Neurosci. 2017;11:296 doi: 10.3389/fncel.2017.00296 29018332 10.3389/fncel.2017.00296 PMC5615225

[37] Zarate MA, Chang EI, Antolic A, Wood CE. Ketamine modulates fetal hemodynamic and endocrine responses to umbilical cord occlusion. Physiol Rep. 2016 9;4(17). 10.14814/phy2.12962 PMC5027363 27597770

[38] Bennet L, Westgate JA, Liu Y-CJ, Wassink G, Gunn AJ. Fetal acidosis and hypotension during repeated umbilical cord occlusions are associated with enhanced chemoreflex responses in near-term fetal sheep. J Appl Physiol Bethesda Md 1985. 2005 10;99(4):1477–82. 10.1152/japplphysiol.00431.2005 15976361

[39] Prout AP, Frasch MG, Veldhuizen RAW, Hammond R, Ross MG, Richardson BS. Systemic and cerebral inflammatory response to umbilical cord occlusions with worsening acidosis in the ovine fetus. Am J Obstet Gynecol. 2010 1;202(1):82.e1–82.e9. 19889382 10.1016/j.ajog.2009.08.020

